# Metabolic reprogramming in neurodegenerative diseases: New insights into mTOR-mediated microglial polarization

**DOI:** 10.1515/jtim-2025-0059

**Published:** 2025-11-25

**Authors:** Wenjing Song, Ying Zhang, Tiance Xu, Tianyi Fan, Jincheng Li, Meiqing Liu, Wenshuo Wei, Xiucong Pei, Runhui Li

**Affiliations:** School of Public Health, Shenyang Medical College, Shenyang, Liaoning Province, China; Key Laboratory of Food Safety and Risk Assessment of Shenyang, Shenyang Medical College, Shenyang, Liaoning Province, China; Key Laboratory of Chronic Disease Assessment and Health Intervention of Shenyang, Shenyang Medical College, Shenyang, Liaoning Province, China; Department of Neurology, Central Hospital Affiliated to Shenyang Medical College, Shenyang, Liaoning Province, China

## To the editor

**Figure 1:**
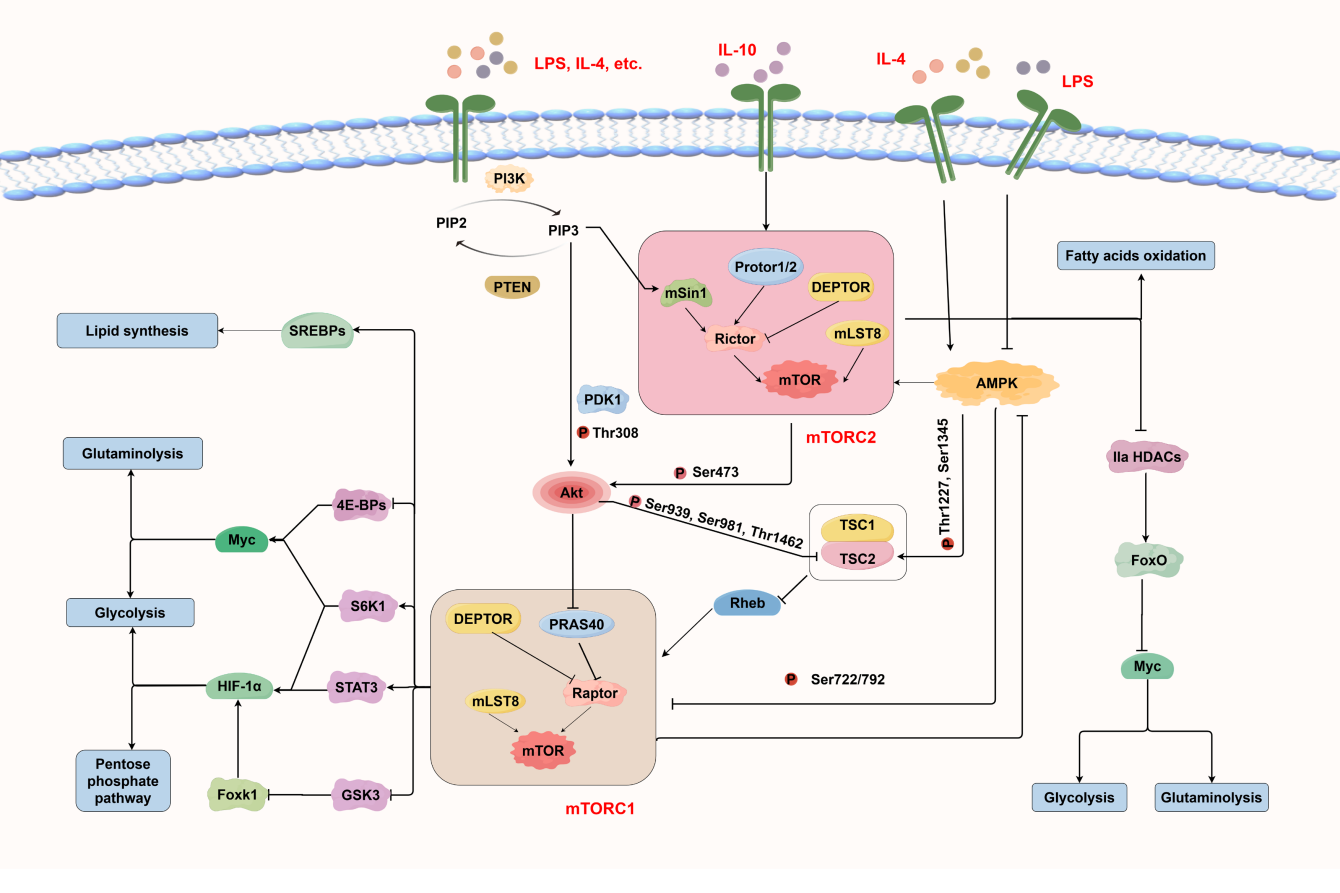
mTORC1 and mTORC2 signaling in microglial metabolic reprogramming. mTORC1: mechanistic target of rapamycin complex 1.

Neurodegenerative diseases (NDs), characterized by significant pathophysiological changes in numerous parts of the central nervous system (CNS), pose substantial challenges to global public health due to the lack of effective treatments to reverse neurodegeneration. Microglia-mediated neuroinflammation is a key contributor to disease progression. Throughout different stages of NDs, microglia polarize into M1 or M2 phenotypes, a process driven by metabolic reprogramming. M1 microglia prefer glycolysis and produce pro-inflammatory cytokines, which may exacerbate neuronal damage. M2 microglia rely more heavily on oxidative phosphorylation (OXPHOS) and fatty acid oxidation (FAO), exerting anti-inflammatory and neuroprotective effects. The mechanistic target of rapamycin (mTOR), which regulates both cellular metabolism and neuroinflammation in microglia, plays a crucial role in NDs. mTOR forms two complexes, mTOR Complex 1 (mTORC1) and mTORC2, each with unique structures and functions (Figure 1). Rapamycin (RAPA) directly inhibits mTORC1 activation but not mTORC2. However, prolonged RAPA treatment can impair mTORC2 function. Interestingly, short-term mTORC1 inhibition by RAPA may inadvertently activate mTORC2.^[1]^ This letter synthesizes current knowledge on mTOR signaling-mediated metabolic alterations and microglial polarization. It outlines how aberrant mTOR signaling contributes to the pathogenesis of NDs and explores potential neurorestorative mechanisms that target this pathway. The text highlights the importance of treatment timing and clinical translation, providing new perspectives for therapeutic strategy development for NDs.

Microglia undergo metabolic programming involving glycolysis, the pentose phosphate pathway (PPP), lipid metabolism, and amino acid metabolism, enabling them to adapt their bioenergetic and biosynthetic activities to microenvironmental signals (Supplementary Figure S1). mTORC1 and mTORC2 coordinate these processes with other key signaling pathways, including the phosphatidylinositol-3 kinase (PI3K)/protein kinase B (Akt) and adenosine monophosphate (AMP)-activated protein kinase (AMPK) pathways, to regulate microglial polarization. When M1 microglia are activated by lipopolysaccharides (LPS) and interleukin (IL)-4, PI3K is stimulated and converts phosphatidylinositol 4, 5 - bisphosphate (PIP2) to phosphatidylinositol 3,4,5-trisphosphate (PIP3). PIP3 activates mTORC2, which subsequently activates Akt fully, while PIP3 also exerts a partial regulatory effect on Akt. Akt subsequently phosphorylates tuberous sclerosis complex protein 2 (TSC2) within TSC1/TSC2 complex, relieving its inhibition of Ras homolog enriched in brain (Rheb) and thereby triggering mTORC1 activation. It also phosphorylates proline-rich akt substrate 40 kDa (PRAS40), causing its dissociation from regulatory-associated protein of mTOR (Raptor) and further promoting mTORC1 activation. Hypoxia-inducible factor 1 alpha (HIF-1α) and Myc serve as major downstream effectors of mTORC1. HIF-1α shifts cellular metabolism toward aerobic glycolysis and reduces mitochondrial OXPHOS, which enhances the production of pro-inflammatory cytokines and reactive oxygen species (ROS). This increased glycolysis subsequently drives the PPP. Myc stimulates both glycolysis and glutaminolysis. Furthermore, mTORC2 regulates Myc expression by phosphorylating class IIa histone deacetylases (HDACs), thereby promoting glycolysis and glutaminolysis. In addition to PI3K/Akt, AMPK negatively regulates mTORC1 to maintain a balance between anabolic and catabolic processes.

PI3K/Akt-mTORC1 and mTORC2-Akt pathways also promote M2 polarization in microglia upon stimulation by IL-4 and IL-10, thereby increasing FAO and OXPHOS. This finding does not contradict the previously described role of Akt in M1 microglial activation, as mammals express three Akt isoforms: Akt1, Akt2, and Akt3. Only Akt1 and Akt2 are present in microglia, with Akt1 primarily promoting M2 polarization and Akt2 mainly inducing M1 polarization.^[2]^

Dysregulated mTOR signaling in microglia disrupts the metabolic flexibility in the CNS, thereby promoting neuroinflammation and neurodegeneration. In Alzheimer’s disease (AD), the hyperactivation of mTOR, specifically the mTORC1 complex, promotes the deposition of amyloid-beta (Aβ), the phosphorylation of tau, and the formation of neurofibrillary tangles. Inhibiting mTOR signaling reduces these pathological features, improves neuronal survival and enhances cognitive function through the promotion of autophagy. During acute inflammatory phases, microglial metabolism shifts substantially from OXPHOS to aerobic glycolysis through the PI3K/Akt-mTORC1 and AMPK-mTORC1 pathways. Chronic Aβ exposure, however, suppresses both OXPHOS and aerobic glycolysis. Furthermore, AD is marked by defects in branched-chain amino acid (BCAA) catabolism, which elevates BCAA levels and promotes tau phosphorylation *via* an mTOR-dependent mechanism.^[3]^ mTORC2 plays distinct roles in AD pathology. Deleting regulatory-associated protein of mTOR (Raptor), but not Raptor, rescues AD-like behaviors and normalizes metabolic dysfunction in phosphatase and tensin homolog (PTEN)-deficient mice.^[4]^ Conversely, stimulating mTORC2 appears neuroprotective. For example, Rictor overexpression in a rodent AD model fully reversed the pathological effects of Aβ on primary neuronal cultures.^[5]^

In Parkinson’s disease (PD), elevated mTOR activity in microglia promotes a shift toward pro-inflammatory states, thereby exacerbating alpha-synuclein (α-Syn) aggregation. This inflammatory transition is accompanied by metabolic reprogramming and mitochondrial dysfunction mediated by the PI3K/Akt or AMPK pathways, similar to findings in AD. For example, in a PD model featuring upregulation of deoxyribonucleic acid (DNA) damage responses 1 (REDD1), which negatively regulates mTORC1, restoration of mTORC1 signaling exerts a neuroprotective effect.^[6]^

In Huntington’s Disease (HD) models, metabolic alterations encompass mitochondrial dysfunction and disrupted cholesterol metabolism.^[7]^ Autophagy is also impaired, resulting in the accumulation of mutant Huntingtin (HTT) protein. Activating mTORC1 alleviates these abnormalities and enhances autophagy, indicating a potential neuroprotective effect.

Pharmacological inhibition to fine-tuning mTOR signaling restores microglial metabolic flexibility and alleviates neuroinflammation, indicating a promising therapeutic strategy for NDs. Current approaches primarily employ rapalogs such as RAPA and its analogs as well as novel adenosine triphosphate (ATP)-competitive inhibitors, each presenting distinct advantages and constraints.^[8]^ In animal models of AD, RAPA delays the aging process, improves cognition, and reduces amyloid oligomers. However, The modest effect on amyloid pathology implies that earlier intervention could yield greater benefits. In a mouse model of mitochondrial permeability transition pore (MPTP)-induced PD, RAPA mitigates symptoms, preserves dopamine neurons, and curbs α-Syn aggregation *via* mTOR pathway inhibition. Although animal studies demonstrate considerable efficacy, RAPA has not yet been validated in ND patients. A single-site open-label phase I clinical trial (NCT04200911) has recently been initiated in humans.^[9]^ Moreover, ATP-competitive mTOR inhibitors target the catalytic site of mTOR, thereby inhibiting both mTORC1 and mTORC2 and achieving a broader blockade of mTOR signaling. In certain NDs, such as AD, mTORC2 dysregulation contributes to pathology. These inhibitors can therefore directly counteract the effects mediated by mTORC2. Other mTOR inhibitors, such as transient receptor potential vanilloid 1 (TRPV1) agonists, immunometabolic reprogramming nanomodulators, and capsaicin (Supplementary Table S1), are currently under preclinical investigation for treating NDs.

A precisely timed therapeutic strategy is essential to achieve neuroprotection while avoiding adverse effects.^[10]^ Inhibition of mTORC1 can promote autophagy and prevent widespread neuronal damage during initial disease stages. For example, reducing mTORC1 is beneficial in early AD by promoting the clearance of protein aggregates. As NDs advance, however, microglia undergo severe metabolic stress. Chronic or late-stage mTORC1 inhibition becomes detrimental because microglia require anabolic activity for repair and maintenance. Additionally, mTORC1 and mTORC2 interact through complex crosstalk and feedback loops, meaning that inhibiting one inevitably affects the other. Nonspecific or poorly timed intervention risks disrupting the balance, potentially leading to side effects such as infection and immunologic derangement.

In summary, mTORC1 and mTORC2 serve as central signaling hubs in microglia, coordinating metabolic reprogramming, immune responses, and cellular maintenance. mTORC1 drives metabolic reprogramming towards glycolysis, PPP, and glutaminolysis, which are particularly relevant for M1 microglial activation. In contrast, mTORC2 supports adaptive metabolic shifts that favor OXPHOS and FAO, which are crucial for M2 microglial functions. Dysregulated mTOR-mediated metabolic reprogramming in microglia contributes to the pathology of NDs such as AD, PD, and HD. Although mTOR inhibitors like RAPA and its analogs show therapeutic promise in preclinical studies, translating these findings into clinical therapies for NDs remains challenging due to the complexity of mTOR signaling.

Future research should elucidate the precise molecular mechanisms by which mTOR complexes regulate microglial metabolic reprogramming in different NDs. Such insight will facilitate the development of selective modulators targeting mTORC1, mTORC2, or their downstream effectors, thereby advancing disease-specific therapeutic strategies. Given that mTOR’s functions are dynamic and context-dependent, defining the optimal intervention windows is crucial to avoid chronic side effects. Another challenge lies in the lack of reliable biomarkers for monitor therapeutic outcomes. Consequently, further research should also focus on the identification and validation of biomarkers to enable personalized and effective therapies.

## Supplementary Information

Supplementary materials are only available at the official site of the journal (www.intern-med.com).

## Supplementary Material

Supplementary Material Details
