# Supplementary material for: Metabolic reprogramming in neurodegenerative diseases: New insights into mTOR-mediated microglial polarization
Source: J Transl Int Med. 2025 Nov 25;13(6):610–3. doi: 10.1515/jtim-2025-0059 (PMC12721358; doi:10.1515/jtim-2025-0059)
Supplement: Supplementary file 1 — Supplementary Material Details [file jtim-2025-0059_sm.pdf]

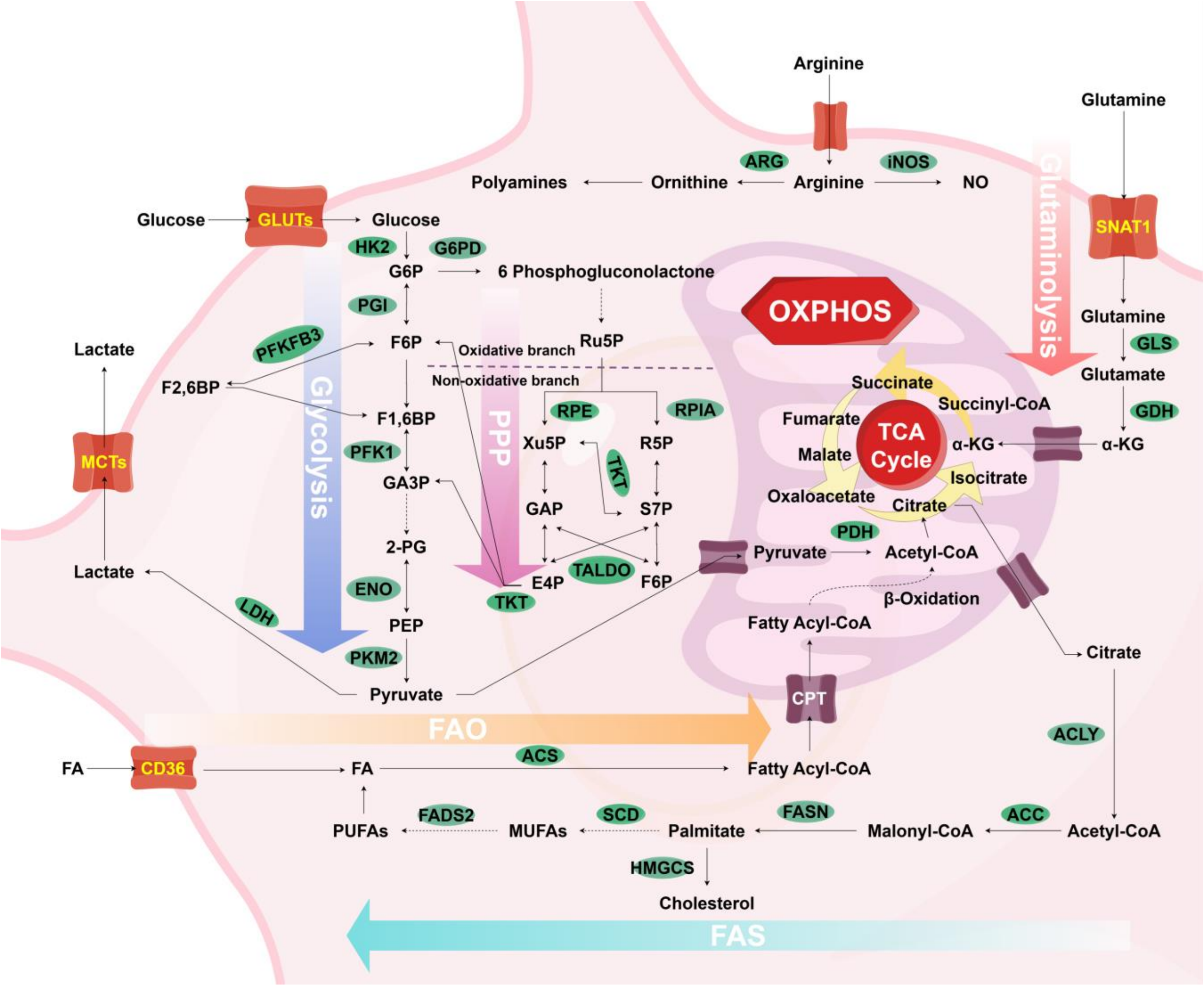

Figure S1: A summary of the major metabolic pathways in microglia.

Table S1: Effects of substances on mTOR-mediated metabolic reprogramming in NDs.

**Substances**	**Diseases**	**Signaling pathways**	**Effects**	**Ref.**
Huangshaodan	AD	mTOR-HIF-1α	Inhibits glycolysis	Shang C *et al.*, Front Pharmacol 2024;15:1434568.
INF-γ	AD	mTOR-HIF-1α	Reverses the defective glycolytic metabolism and inflammatory functions of microglia	Wang Y *et al.*, Int Immunopharmacol 2023;115:109691.
TRPV1 agonist	AD	Akt-mTOR-HIF-1α	Enhances OXPHOS, Inhibits glycolysis	Li J *et al.*, EMBO Rep, 2021;22:e52013.
TREM2-activating antibody	AD	AMPK-mTOR	Inhibits glycolysis	Van Lengerich B *et al.*, Nat Neurosci 2023;26:416.

Immunometabolic reprogramming nanomodulators (GAF NPs)	AD	Akt-mTOR-HIF-1α	Shifts from glycolysis to OXPHOS	Yang F *et al*., ACS Nano 2023;17:15724.
Erjingpill bionic cerebrospinal fluid	AD	PI3K/Akt-mTOR	Inhibits glycolysis	Wang S *et al*., J Ethnopharmacol 2024;333:118412.
α-Mangostin	PD	AMPK-mTOR	Shifts from glycolysis to OXPHOS	Wang D *et al*., Acta Pharm Sin B 2023;13:834851.
Capsaicin	PD	Akt-mTOR-HIF-1α	Inhibits glycolysis, enhances OXPHOS	Lu J *et al*., J Neuroinflammation 2022;19:113.
Glycolysis inhibitors (2-DG) and 3-bromopyruvic acid (3-BPA)	PD	AMPK-mTOR	Inhibits glycolysis	Cheng J *et al*., J Neuroinflammation 2021;18:129.
Levistilide A	PD	AMPK-mTOR	Inhibits glycolysis	Zhang M *et al*., Molecules 2024;29:912.

NDs: neurodegenerative diseases; INF-γ: interferon-γ; TRPV1: transient receptor potential vanilloid 1; TREM2: triggering receptor expressed on myeloid cells 2; GAF NPs: gold nanocages (Au NCs) surface-functionalized with glutathione (GSH) and loaded with the immunosuppressant fingolimod hydrochloride (FTY720); AD: Alzheimer's disease; PD: Parkinson's disease; mTOR-HIF-1α: mechanistic target of rapamycin- Hypoxia-inducible factor 1 alpha; Akt: protein kinase B; AMPK: adenosine monophosphate-activated protein kinase; PI3K: phosphatidylinositol-3 kinase; OXPHOS: oxidative phosphorylation.
